# Supplementary material for: Personal and General Views on Aging, Non-Communicable Diseases, and Their Interaction as Cross-Sectional Correlates of Vigorous Physical Activity in UK Individuals Aged 50+
Source: Healthcare (Basel). 2025 Aug 21;13(16):2071. doi: 10.3390/healthcare13162071 (PMC12386382; doi:10.3390/healthcare13162071)
Supplement: Supplementary file 1 [file healthcare-13-02071-s001.zip › healthcare-3761046-supplementary.pdf]

Supplementary Table S1. Regression model for the cross-sectional association of awareness of age-related gains with vigorous physical activity.

Supplementary Table 1.

	Univariable model			Multivariable model		
**Cross-sectional predictors**	**OR (95% CI)**	**p-value**	**Pseudo R^2**	**OR (95% CI)**	**p-value**	**Pseudo R^2**
Awareness of age-related gains	1.07 (1.05; 1.09)	.001	0.01	1.06 (1.04; 1.08)	.001	0.01
Age				0.99 (0.98; 1.01)	.305	0.0004
Sex				1.19 (0.98; 1.45)	.072	0.002
Education						
Primary or secondary education				0.68 (0.52; 0.89)	.006	0.004
Post-secondary education				0.62 (0.47; 0.81)	.001	0.01
Vocational qualification				0.90 (0.72; 1.12)	.337	0.0004
University degree				Reference category		
Marital status						
Married or civil partnership				Reference category		
Widowed				0.93 (0.67; 1.28)	.660	0.0001
Separated/divorced				1.00 (0.76; 1.32)	.982	0.00
Co-habiting				1.04 (0.71; 1.53)	.823	0.00
Single				0.82 (0.60; 1.14)	.245	0.001
Working status						
Employed				0.94 (0.74; 1.18)	.581	0.0001
Retired				Reference category		
Number of non-communicable diseases				0.92 (0.85; 0.99)	.020	0.004

*OR= Odds ratio. Pseudo R^2= Pseaudo coefficient of determination.

Supplementary Table S2. Regression model for the cross-sectional association of awareness of age-related losses with vigorous physical activity.

Supplementary Table 2.

	Univariable model			Multivariable model		
**Cross-sectional predictors**	**OR (95% CI)**	**p-value**	**Pseudo R^2**	**OR (95% CI)**	**p-value**	**Pseudo R^2**
Awareness of age-related losses	0.88 (0.86; 0.90)	.001	0.02	0.88 (0.86; 0.91)	.001	0.02
Age				0.99 (0.98; 1.01)	.380	0.0003
Sex				1.16 (0.95; 1.41)	.132	0.002
Education						
Primary or secondary education				0.75 (0.57; 0.99)	.043	0.002
Post-secondary education				0.66 (0.50; 0.87)	.003	0.01
Vocational qualification				0.91 (0.73; 1.14)	.409	0.0003
University degree				Reference category		
Marital status						
Married or civil partnership				Reference category		
Widowed				1.07 (0.78; 1.48)	.664	0.0001
Separated/divorced				1.04 (0.79; 1.37)	.775	0.0001
Co-habiting				0.98 (0.67; 1.44)	.928	0.00
Single				0.77 (0.56; 1.07)	.121	0.001
Working status						
Employed				0.85 (0.68; 1.07)	.172	0.001
Retired				Reference category		
Number of non-communicable diseases				0.99 (0.92; 1.07)	.891	0.0001

*OR= Odds ratio. Pseudo R^2= Pseaudo coefficient of determination.

Supplementary Table S3. Regression model for the cross-sectional association of expectations regarding aging with vigorous physical activity.

Supplementary Table 3.

	Univariable model			Multivariable model		
**Cross-sectional predictors**	**OR (95% CI)**	**p-value**	**Pseudo R^2**	**OR (95% CI)**	**p-value**	**Pseudo R^2**
Expectations regarding aging	1.05 (1.03; 1.07)	.001	0.01	1.05 (1.03; 1.07)	.001	0.01
Age				0.99 (0.98; 1.004)	.187	0.001
Sex				1.13 (0.93; 1.37)	.230	0.001
Education						
Primary or secondary education				0.76 (0.58; 1.01)	.055	0.002
Post-secondary education				0.65 (0.49; 0.86)	.003	0.01
Vocational qualification				0.94 (0.75; 1.18)	.612	0.0001
University degree				Reference category		
Marital status						
Married or civil partnership				Reference category		
Widowed				0.97 (0.70; 1.33)	.838	0.00
Separated/divorced				1.00 (0.76; 1.32)	.996	0.00
Co-habiting				1.00 (0.68; 1.47)	.988	0.00
Single				0.74 (0.54; 1.03)	.073	0.002
Working status						
Employed				0.91 (0.72; 1.14)	.403	0.0003
Retired				Reference category		
Number of non-communicable diseases				0.93 (0.87; 1.00)	.058	0.002

*OR= Odds ratio. Pseudo R^2= Pseaudo coefficient of determination.

Supplementary Table S4. Regression model for the cross-sectional association of number of non-communicable diseases with vigorous physical activity.

Supplementary Table 4.

	Univariable model			Multivariable model		
**Cross-sectional predictors**	**OR (95% CI)**	**p-value**	**Pseudo R^2**	**OR (95% CI)**	**p-value**	**Pseudo R^2**
Number of non-communicable diseases	0.90 (0.84; 0.96)	.002	0.002	0.91 (0.85; 0.98)	.015	0.004
Age				0.99 (0.98; 1.00)	.137	0.001
Sex				1.23 (1.02; 1.50)	.034	0.003
Education						
Primary or secondary education				0.60 (0.52; 0.90)	.007	0.004
Post-secondary education				0.62 (0.47; 0.82)	.001	0.007
Vocational qualification				0.91 (0.73; 1.14)	.436	0.0003
University degree				Reference category		
Marital status						
Married or civil partnership				Reference category		
Widowed				0.98 (0.71; 1.35)	.885	0.00
Separated/divorced				1.02 (0.78; 1.35)	.872	0.00
Co-habiting				1.04 (0.71; 1.53)	.836	0.00
Single				0.78 (0.56; 1.07)	.127	0.002
Working status						
Employed				0.90 (0.72; 1.13)	.387	0.0003
Retired				Reference category		

*OR= Odds ratio. Pseudo R^2= Pseaudo coefficient of determination.
